# Filling Labial Surfaces of Upper Incisiors

**Published:** 1857-09

**Authors:** A. J. Volck


### Miscellaneous.

### FILLING LABIAL SURFACES OF UPPER INCISORS.

BY A. J. VOLCK, D. D. S.

I beg leave to submit to the profession the description of an operation, which, having been suggested by Professor Maynard, of Washington, I have tried in a number of cases and found of such importance, that I believe its publication in your valuable Journal will be acceptable to the careful operators of the dental profession.

This operation consists in setting a piece of enamel in the cavity of a decayed tooth, when such a cavity is exposed to the sight, thereby avoiding the ungainly appearance of gold fillings in the front of the mouth. The only cases in which I have as yet applied this method were in cavities in the anterior surface of front teeth. It is done by fitting a piece of enamel into the prepared cavity loosely, walling it into the same by a continuous ring of gold foil. The narrower, of course, this gold ring can be made, the more perfect will be the deception. In the first operation of this kind that I attempted the width of the gold ring is about equal to the thickness of a five cent piece; even in this case the improvement over the large gold filling which had previously disfigured the tooth, is striking, and made me hope for much satisfaction from future practice. Having since become more adroit in this operation, I have accomplished fillings in which the rim of gold is not thicker than a small main spring. These fillings are barely preceptible to any but the closest observer.

In preparing the cavity for this purpose pains are taken to have the walls perpendicular and the bottom of it perfectly even and flat, avoiding if possible rounded edges, which would make the gold ring appear thicker than it actually is. In cases where the cavity is deep it may be partly plugged in the common manner, and a bed thus formed on the bottom of the cavity for the reception of the enamel. The enamel must be fitted so as to correspond precisely with the shape of the cavity, having also perpendicular sides and allowing sufficient room between it and the walls of the cavity fôr the filling in of the gold. Around the sides of this patch of enamel a small, barely perceptible, groove can be cut with a sharp file as an additional security for the stability of the gold filling. To fasten this enamel patch in the cavity, my way has been, to wrap around it a strip of No. 4 gold foil of sufficient thickness to fill up the space between it and the sides of the cavity, leaving the strip wider than the enamel so as to allow for condensing and finishing the plug; this I passed gently into the cavity, assisting on all sides with a small and very thin plugger, so as to make the gold arrive with the enamel at the bottom of the cavity—after it has been inserted in this manner, the gold plug can be condensed and finished as any common filling. The best material for the enamel will be found to be the porcelain of which artificial teeth are made—a piece of common plate tooth of the proper color has answered to perfection. This enamel is easily finished down with corundum slabs and polished with an Arkansas stone.

I have taken occasion to show some of these fillings to prominent members of the profession, and have been gratified by their approval and encouragement. There is no reason why these plugs, if properly done, should not stand the test of time like any other good filling, and there can certainly be nothing more gratifying than their appearance.

In many cases gold fillings in the most prominent part of the mouth disfigures an otherwise beautiful face, and the time and care expended in this operation will amply repay a careful operator in the satisfaction and gratitude of his patients.—*Am. Journal of Dental Science.*

